# Clinical applications of artificial intelligence and radiomics in neuro-oncology imaging

**DOI:** 10.1186/s13244-021-01102-6

**Published:** 2021-10-21

**Authors:** Ahmed Abdel Khalek Abdel Razek, Ahmed Alksas, Mohamed Shehata, Amr AbdelKhalek, Khaled Abdel Baky, Ayman El-Baz, Eman Helmy

**Affiliations:** 1 Department of Diagnostic Radiology, Faculty of Medicine, Mansoura University, Elgomheryia Street, Mansoura, 3512 Egypt; 2 Biomaging Lab, Department of Bioengineering, University of Louisville, Louisville, KY 40292 USA; 3 Internship at Mansoura University Hospital, Mansoura Faculty of Medicine, Mansoura, Egypt; 4 Department of Diagnostic Radiology, Faculty of Medicine, Port Said University, Port Said, Egypt

**Keywords:** Artificial intelligence, Machine learning, Deep learning, Glioma, Radiomics

## Abstract

This article is a comprehensive review of the basic background, technique, and clinical applications of artificial intelligence (AI) and radiomics in the field of neuro-oncology. A variety of AI and radiomics utilized conventional and advanced techniques to differentiate brain tumors from non-neoplastic lesions such as inflammatory and demyelinating brain lesions. It is used in the diagnosis of gliomas and discrimination of gliomas from lymphomas and metastasis. Also, semiautomated and automated tumor segmentation has been developed for radiotherapy planning and follow-up. It has a role in the grading, prediction of treatment response, and prognosis of gliomas. Radiogenomics allowed the connection of the imaging phenotype of the tumor to its molecular environment. In addition, AI is applied for the assessment of extra-axial brain tumors and pediatric tumors with high performance in tumor detection, classification, and stratification of patient’s prognoses.

## Key points

- AI methods utilized conventional and advanced techniques to differentiate brain tumors from non-neoplastic lesions.
- AI used in the diagnosis of gliomas and discrimination of gliomas from lymphomas and metastasis.
- AI has a role in the grading, prediction of treatment response, and prognosis of gliomas.
- Radiogenomics allowed the connection of the imaging phenotype of the tumor to its molecular environment.
- AI is applied for the assessment of extra-axial brain tumors and pediatric tumors.

## Introduction and background

### Brain tumors

The World Health Organization (WHO) has provided an update of brain tumor classification in 2016 incorporating genetic information. Discrimination between different types of brain tumors is problematic at imaging. Accurate diagnosis is crucial for planning of treatment to improve patient’s outcome, helpful in the grading of tumors and response after therapy [1–7]. Brain tumor biopsy is considered the gold standard for diagnosis. However, it carries the risk of procedure-related complications in about 6% of cases [2, 3, 8, 9].

### Methods of examination

Conventional MR imaging relies particularly on overall tumor morphology, composition, location, mass effect, and multiplicity. Some limitations remain as challenges such as the differentiation of brain tumors from simulating lesions, tumor characterization and grading, and the discrimination of recurrent tumors from tissue necrosis. Advanced MR imaging including diffusion, diffusion tensor imaging (DTI), perfusion MR techniques as arterial spin labeling (ASL), dynamic susceptibility contrast (DSC), dynamic contrast-enhanced imaging (DCE), and MR spectroscopy technique are quantitative biomarkers used to determine tumor morphology and function [10–14]. The emerging Artificial Intelligence (AI) methods have shown significant progress in the field of radiological-based medical imaging applications. The basic concept of AI refers to any method that allows human intelligence to be imitated by computers [2, 4]. Machine learning (ML) is a subset of AI techniques that utilize algorithms that evolve as new data are introduced. Deep learning (DL) is a subclass of ML-based on neural networks, applying a large number of layers, and allowing more complex classification processes [1–5].

Both radiology and computing have advanced to the point that Artificial Intelligence (AI) can be applied to radiology problems: radiology has gone digital, with all data stored in radiology information system picture archives and communication system archives; AI has evolved to the point where automated image processing is possible. Increased computing capacity, data storage with low cost, and faster rates of transferring data have all contributed to this growth. In recent years, this has culminated in a large rise in publications on AI in radiology, regarding a vast variety of potential applications including identification, segmentation, classification, and outcome prediction.

This article addresses the fundamentals, existing workflow, and methods used in AI-based radiological applications in the medical field. Additionally, we aim to review the clinical applications of AI in brain tumors.

### Artificial intelligence (AI)

Artificial Intelligence (AI) refers to the computation ability to perform tasks that are similar to those performed by humans in order to highly utilize unique inputs generating outputs with high added value. Medical imaging is actually one of the most exciting applications of AI right now. Radiologists can make great use of computers with routine detection and diagnosis tasks. The aim of encouraging the use of Computer-Aided Diagnostic (CAD) systems using the state-of-the-art AI techniques was to assist radiologists in the detection and analysis of potential lesions which in turn enables distinguishing between lesions, reducing errors, and increasing radiological efficiency. As a result, there have been continuous and incremental efforts to enhance AI's diagnostic efficiency to be promoted for everyday clinical practice [15]. The invention of artificial neural networks (ANN) in the middle of the last century and their subsequent evolution, which introduced the principles of computational learning models, ML, and DL, is largely responsible for the growth of AI.

### Machine learning (ML)

Applications of ML necessitate a collection of pathological data as input that the machine will use for self-training, and such data should always provide the desired output to be expected. Whether the input was previously labeled by human experts or whether the computer performed direct data extraction using a variety of computational methods identifies two types of ML; supervised or unsupervised [16]. The optimal ML model must include the features that are most important to the outcome (local features) as well as the most generic ones (global features) with the ability of generalization for new unseen inputs.

### Deep learning (DL)

Deep learning (DL) allows more complex classification processes as well as automatic dimensionality reduction through a hierarchical feature extraction criterion. Using convolutional neural networks (CNN) and the inclusion of multiple neural layers between input and output, enhance the robustness of DL and provide the ability to replicate the human brain processes in the training phase. DL is a hot subject of research that has practically exploded in recent years. By combining ML/DL image processing with clinical and, where appropriate, pathological/histological data, the ability to relate fundamental diagnostic patterns and features of radiological scans (with different modalities) to a particular pathological and histological subtyping has created a new field in research developing which is Radiomics [17].

### Radiomics

**Fig. 1:**
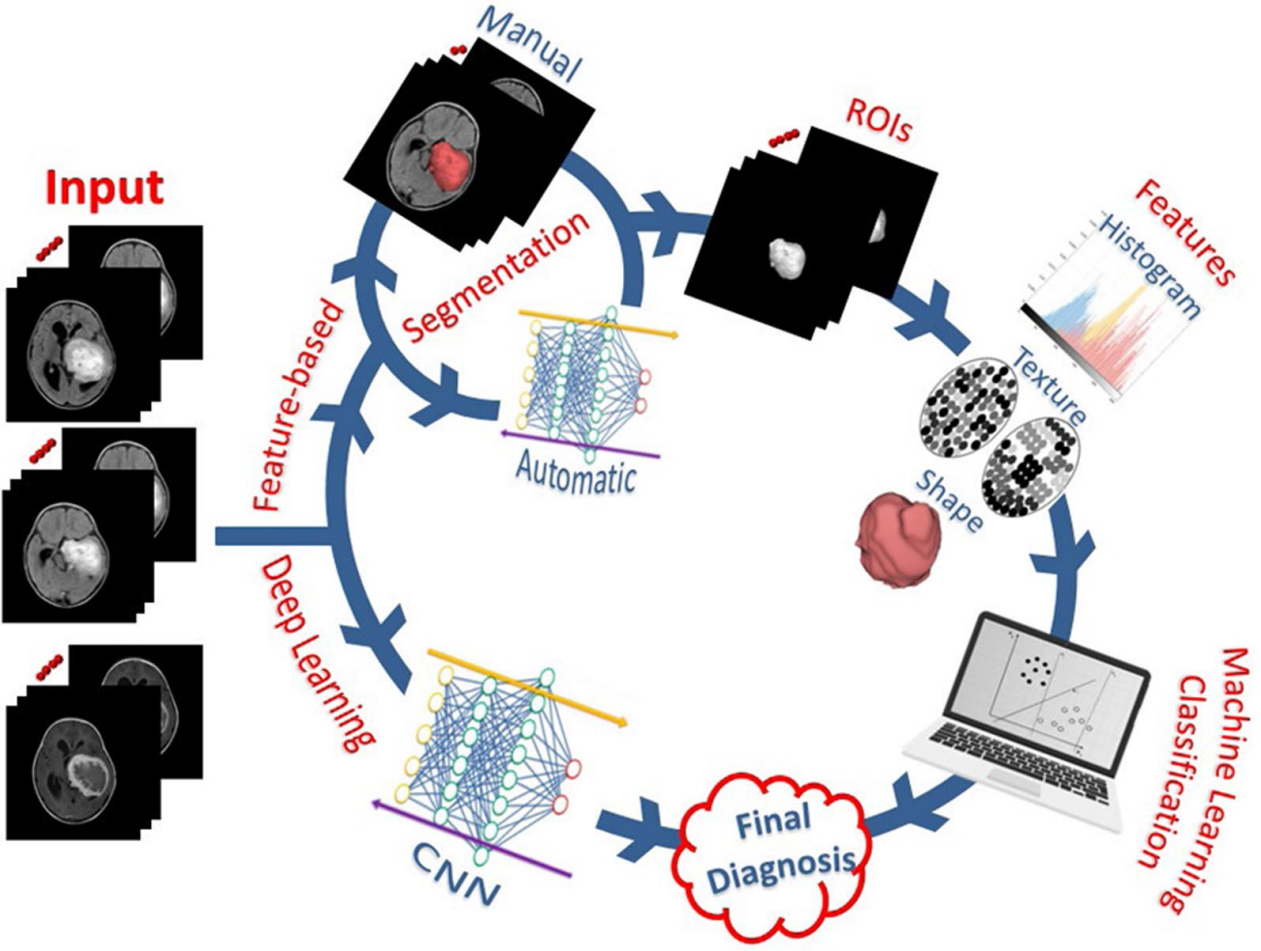
General framework showing the main steps of the radiomics

Radiomics is a new translational field in which a range of attributes, such as geometry, strength, and texture, are determined from radiological images to allow for capturing different imaging patterns. These patterns could be used for tumor subtyping, grading, and staging. In addition, Radiomics is usually used in systems in multiple variations for prediction, prognosis, monitoring, and treatment response assessment [17]. There are two main types of radiomics: feature-based and deep learning-based radiomics. Unlike these clinical evaluations affected by the human reader, the results are more stable, accurate, and reproducible. Thanks to the ability of radiomics features to be calculated using multiple mathematical algorithms (feature-based) or created statistically from ML-based complex computational models during the training phases (deep learning-based) in an automatic process. Figure 1 shows the general framework for radiomics.

#### Feature-based radiomics

Feature-based radiomics works by extracting a set of numerical features from a segmented region/volume of interest (ROI/VOI for 2D/3D). Then, feature selection algorithms evaluate a subset of specific features to prevent overfitting and create robust prediction models. Feature-based radiomics does not necessitate large datasets because the measured features are specified separately from the data in a typically short computation time. Additionally, since these mathematically represented features are established, a biological comparison can be interpreted. However, most of the extracted features are characterized by their complex numerical nature, making the direct interrelation between these numerical features and physiological context difficult, if not impossible, to be achieved by human perception. The following is a list of the most important processing phases in the feature-based radiomics process [18].

**Fig. 2:**
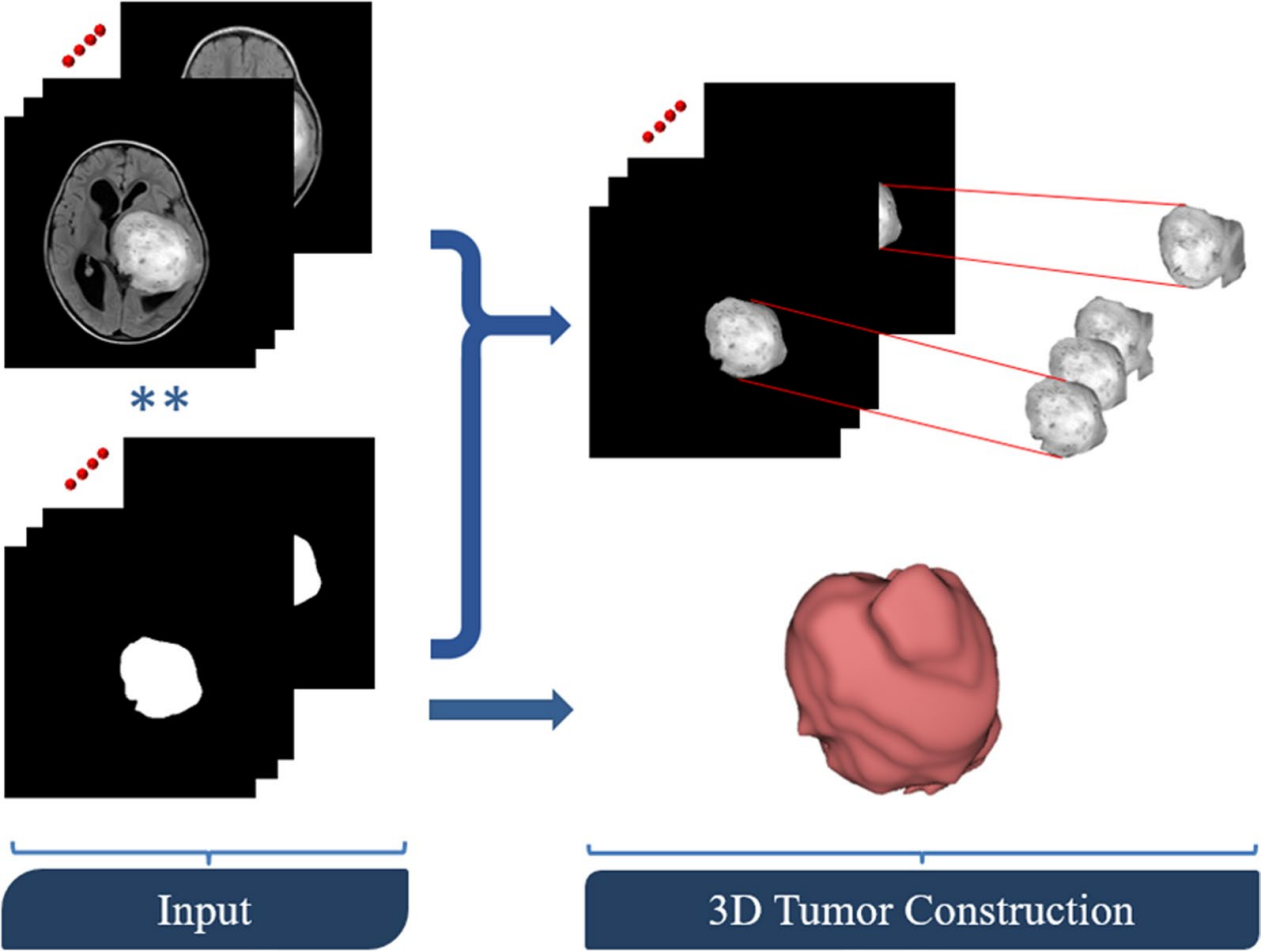
Pre-processing step on glioma tumor subject (T2-FLAIR imaging modality)

**Fig. 3:**
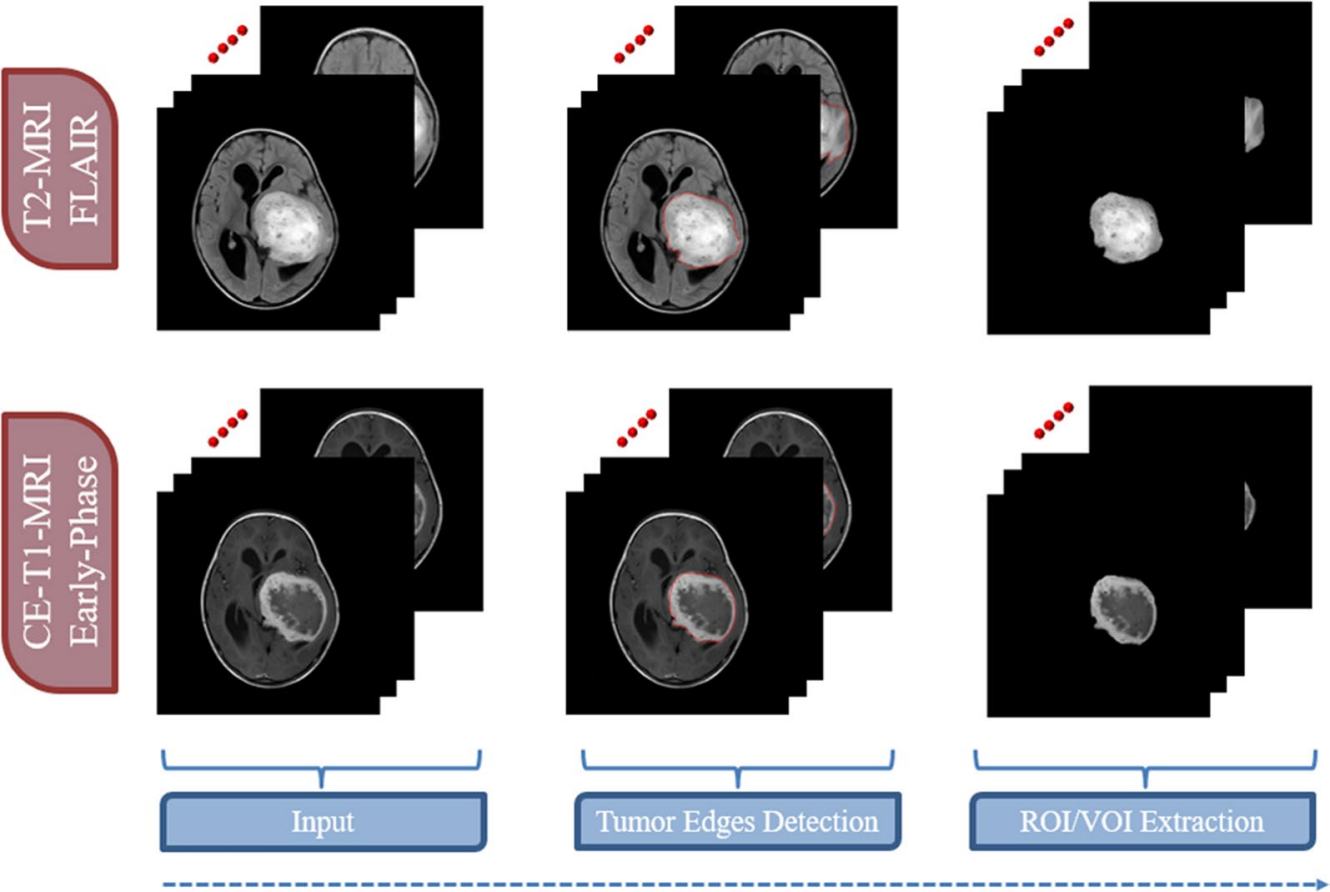
Manual segmentation for a glioma tumor in two different imaging modalities

**Fig. 4:**
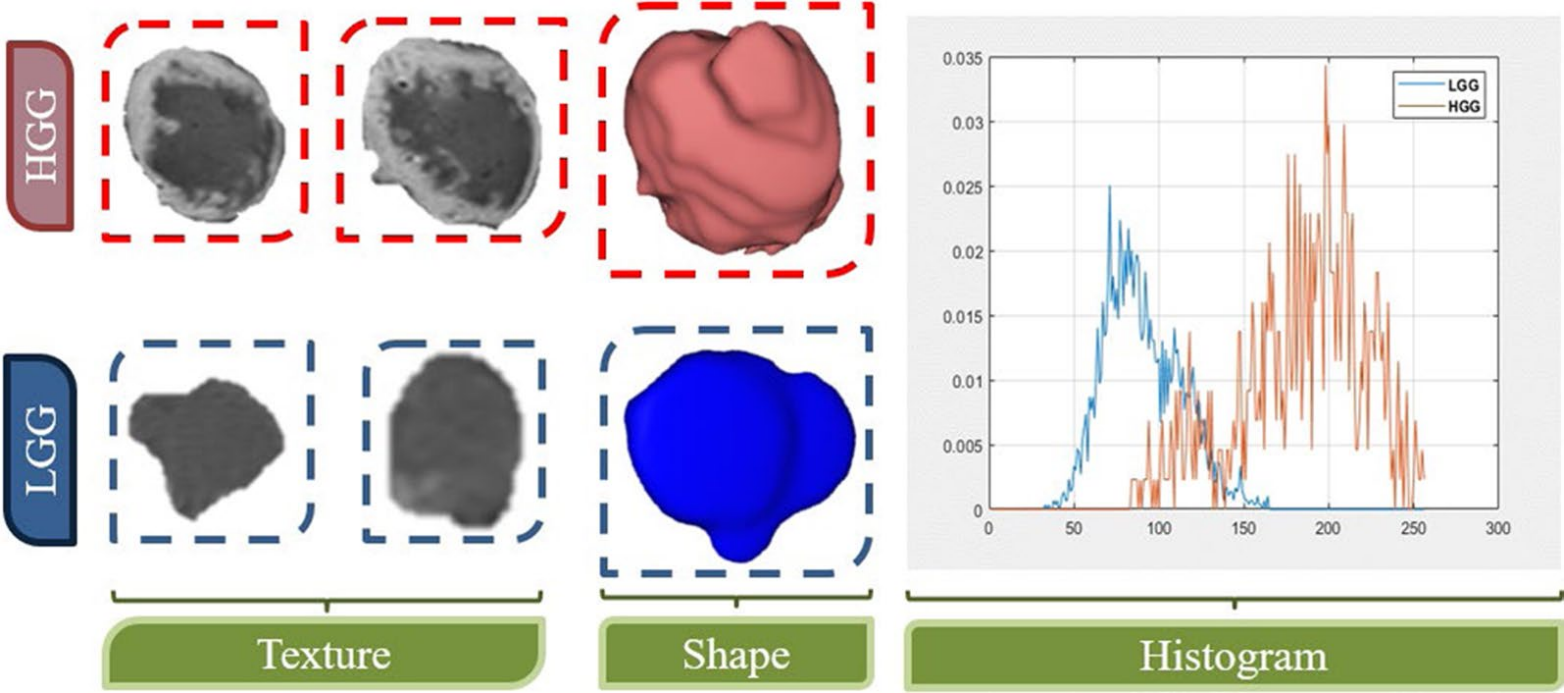
Differences between three groups of features (texture, shape, and histogram) extracted from two glioma subjects with different grades (HGG and LGG)

- *Image pre-processing* Generating quantitative features be in a good shape of repeatability and generalizability from radiological images is the primary aim of radiomics. Several common pre-processing steps must be undertaken in order to accomplish this objective, namely corrections of MRI field inhomogeneities, noise reduction, spatial resampling, spatial smoothing, and intensity normalization. The pre-processing of a Glioma tumor is shown in Fig. 2.
- *Tumor segmentation* Precise segmentation is an essential step toward an accurate radiomics analysis. The manual segmentation of lesions, which involves areas of contrast enhancement, necrosis, and surroundings detection, is a subjective and time-consuming process. To address this, many machine learning algorithms, such as DL-based approaches, are currently being implemented and tested for automatic tumor localization and segmentation. While these methods can now be used to help with tumor segmentation, their efficacy must be demonstrated before they can be used in clinical practice. Figure 3 shows the steps of manual segmentation on a Glioma tumor in two different imaging modalities (CE-MRI at Early Phase and T2-MRI Flair).
- *Feature extraction* Medical images may be used to obtain a number of quantitative characteristics, the majority of which represent tumor heterogeneity. Despite the fact that tons of features can be extracted with different mathematical meanings, features are typically grouped into four subgroups, namely shape characteristics [18], first-order statistics (histogram-based) features [19], second-order statistics (textural) features [20], and higher-order statistics features [21]. Differences between features extracted from two Gliomas with different grades (high-grade HGG and low-grade LGG) are shown in Fig. 4.
- *Feature selection* The extracted quantitative features may not be equally significant. The majority of the features are prone to redundancy, strong correlation and ambiguity which might lead to data overfitting and extreme increment in image noise sensitivity in the dependent predictive models. Running feature selection before learning phases is one way to reduce the chance of these issues. Feature selection strategies take into account the relationship between the features and the class labels, resulting in selecting features regarding their contribution to the classification issue. In other words, these techniques select the features that contribute the most to distinguishing between the classes. In radiomics, the three popular methods are filter methods (univariate methods), wrapper methods (multivariate methods), and embedded approaches [22].
- *Model generation and evaluation* Depending on the purpose of the analysis, many ML-based algorithms can be used to produce predictive models. The Cox proportional hazards model in the case of examined survival data, neural networks, support vector machines (SVM), decision trees (e.g., random forests), linear regression, and logistic regression are the most common algorithms in radiomics. In supervised ML tasks, the used dataset is often divided into training and validation using stratified splitting to ensure that the training and validation datasets roughly maintain the same samples distribution as the classes distribution in the entire data collection. Following the training and validation steps, the model should preferably be applied to a new unseen testing dataset which reflect the data that the model will experience in clinical practice [21–23].

#### Deep learning-based radiomics

Various network architectures or stacks of linear and nonlinear functions, like convolutional neural networks (CNNs) or auto-encoders, are used in deep learning-based radiomics to find the most important/critical characteristics from the radiological images. Without any prior description or collection of features, single-layer neural networks cascaded system is included in the learning process of structures in radiological-based data that are important for classification [23]. This cascading process helps to generate/reduce features gradually to obtain the most dominant/important features. Finally, the previously generated features can be processed further by the network for analysis and classification, or they can exit the network and go through the model generation process using different classifiers such as decision trees, regression models, or support vector machines, similar to the feature-based radiomics method. Techniques like regularization and dropout, fraction are used to prevent overfitting. Deep learning-based radiomics require larger datasets than feature-based radiomics because of the high correlation between the input data and the extracted features, which prevents its applicability in multiple fields of research where the availability of the dataset is restricted such as neuro-oncological research. Transfer learning, on the other hand, is a technique for circumventing this constrain by using pre-trained neural networks that are used for training over a separate, but closely related purpose, for example, a neural network trained with imaging data for automated glioma segmentation may be used for segmenting brain metastases [24]. The amount of data needed to produce reliable performance, as well as the computational demand, is reduced by leveraging the network’s prior knowledge.

The aim of work is to review the clinical applications of AI in brain tumors.

### Clinical applications of AI in brain tumors

Table 1 shows the clinical applications of AI in brain tumors.

**Table 1:** Clinical application of AI in neuro-oncology

*I—Gliomas*
I—Differentiation of neoplastic from non-neoplastic lesions
High-grade gliomas versus tumefactive demyelinating diseases
Gliomas versus inflammation
II—Grading of gliomas
Low versus high grade
Grade II versus grade III
III—Radiogenomics
Glioma
Oligodendroglioma
IV—Pre-treatment evaluation
Tumor segmentation
Infiltration and extent
V—Prognostic value-survival
VI—Post-treatment evaluation
Pseudo-progression
Residual/recurrence versus post-treatment changes
*II—Non glioma*
Metastasis
PCNSL
Hemangioblastoma
*III—Extra-axial brain tumors*
Meningioma
Schwannoma
Pituitary adenoma
*IV—Pediatric brain tumors*
Characterization
Radiogenomics

*PCNSL* primary central nervous system lymphoma

## Radiomics of gliomas

Grading of glioma is classified by the WHO into low grade (I and II) and high grade (III and IV). Glioblastoma multiform (GBM) is a grade IV tumor and is the most common primary brain tumor [25–30]. Distinguishing glioma from non-neoplastic lesions is of utmost importance in the clinical practice as each entity has its different strategy of treatment and prognosis. Determination of glioma grade before therapy is crucial for the optimization of treatment strategy, prediction of therapeutic response, prognosis, and survival [29–34].

### Differentiation of neoplastic from non-neoplastic lesions

#### High-grade gliomas versus tumefactive demyelinating diseases

Tumefactive multiple sclerosis lesions (MS) with atypical features can mimic high-grade gliomas on conventional MR imaging [35]. A study used dynamic texture parameter analysis (DTPA) and extracted features from the first pass of contrast phase of DSC-enhanced perfusion maps and differentiated glioblastomas from tumefactive MS [36].

#### Glioma versus inflammation

Sometimes, MR diagnostic dilemma exists in differentiating atypical cases of inflammation and glioma due to similar radiological features, subjective evaluation, and lack of quantitative indicators [37, 38]. A novel study tried to differentiate between inflammation and grade-II glioma using LASSO algorithms to select features and based on non-contrast imaging features. The study achieved promising results with (AUC) of 0.988 in the primary cohort, and 0.950 in the validation cohort, but the small sample size and retrospective design of the study limit its potential usefulness in differentiating inflammation from glioma [39].

### Grading of gliomas

Several studies discussed the radiomics for glioma grading. A study extracted a large set of radiomics features from routine brain MRI and then used a random forest classifier that yielded a high AUC of 0.92 for evaluation of glioma grade after fivefold cross-validation [40].

#### Low-grade versus high-grade gliomas

A study investigated the role of radiomics in differentiating grade II from grade III and IV; they extracted the radiomics features from conventional, diffusion, and perfusion ASL MRI. An SVM classifier was used and the study showed a high AUC of 0.97and an accuracy of 98%. They concluded also that post-contrast T1 (T1C) is the best single sequence to be compared with multiparametric textural analysis [41]. Another study investigated the conventional and advanced multiparametric MRI (DTI, perfusion, and MR spectroscopy) to derive features to differentiate between low- and high-grade gliomas. Three different SVM classifiers were applied, and the highest performance was achieved with 96% accuracy and an AUC of 0.96. This study was limited mainly by the small patients sample size leading to bias in conducting data analysis, also genomic features would be used to assess their utility [42].

#### Grade II versus grade III gliomas

A study investigated MR textural features derived from DCE MRI in differentiating grade II from grade III gliomas and grade III from grade IV and reported that AUC obtained from entropy textural features was 0.885 and from the inverse different moment (IDM) was 0.901 [43]. Another study extracted radiomics features from conventional MRI and perfusion-weighted imaging using the SVM classifier to differentiate between grade II and III and yielded low classification errors of about 3.7% [44].

### Radiogenomics

#### Glioma radiogenomics

The WHO 2016 classification update of gliomas comprises genetic information for diagnosis. Specific genetic alterations are substantially linked with tumorigenesis. The molecular genotype of the tumor, as well as its histopathology, significantly affects survival. Radiogenomics is an emerging field that refers to the relationship between imaging phenotypes and gene expression patterns, this might allow improved diagnosis, decision-making, and predicting patient outcomes [27, 45, 46]. Important prognostic glioma biomarkers include isocitrate dehydrogenase (IDH), chromosome arms 1p/19q-codeletion, methylguanine-DNA methyltransferase status (MGMT). Combined immunohistochemistry with genome sequencing is a standard method for distinguishing glioma mutations [46, 47]. Table 2 shows radiogenomic features of the most important glioma biomarkers.

**Table 2:** Radiogenomic features of glioma biomarkers

	Glioma biomarker	Features
1	IDH	IDH mutant
		Predominantly hypoenhancing diffuse gliomas
		80% LGG and 10% GBM
		Better survival
		IDH wild type
		Predominantly enhancing gliomas
		90% of GBM
		Worse survival
2	1P/19q codeletion	30% of LGG
		1P/19q codeletion + IDH mutant glioma
		Defines oligodendroglioma
		“poorly circumscribed” margins
		1P/19q non codeletion + / − IDH mutant glioma
		Defines astrocytoma
		“circumscribed” margins
		(T2-FLAIR mismatch) pattern
3	MGMT mutation	33–57% of diffuse glioma patients
		Better prognosis
4	EGFR mutation	40% of glioblastoma patients
		Splice variant (EGFRvIII)
		31% of glioblastoma

*IDH* isocitrate dehydrogenase, *MGMT* methylation of O-6-methylguanine-DNA-methyltransferase, *EGFR* epidermal growth factor receptor

#### IDH mutations

Isocitrate dehydrogenase (IDH) is an enzyme of Krebs cycle, which converts isocitrate to a-ketoglutarate (aKG). Currently, glioma is classified into subtypes based on IDH mutations (IDH 1 and IDH 2) genotype. The mutant type is IDH positive, while the wild-type glioma is IDH negative [25]. Accumulation of alpha-ketoglutarate from isocitrate occurs in wild type, but with IDH mutations, the isocitrate becomes 2-hydroxyglutarate, an oncometabolite that is absent in wild type. Accumulation of 2-hydroxyglutarate can be detected by MR spectroscopy at 7 T with high specificity, but it is not available in most centers which limit its utility [48].

IDH mutant gliomas are associated with improved survival as they demonstrate lower regional cerebral blood flow and volume on perfusion studies, also higher apparent diffusion coefficient (ADC) values on DW-MRI. Predominantly hypoenhancing diffuse gliomas are IDH mutants and comprise about 80% lower-grade gliomas and 10% GBM with better survival. On the other hand, predominantly enhancing gliomas are IDH wild type and comprise 90% of GBM with worse survival [49].

Conventional MRI features that differentiate IDH mutant from IDH wild type includes indistinct margins, T2 hyperintensity of tumor regions but hypointense in FLAIR images (T2-FLAIR mismatch). A retrospective study with a limited sample size applied CNNs to the conventional MR features, showed an accuracy of 92% and demonstrated that IDH wild-type tumors have more infiltrative margins [50]. A more recent study has correlated multiparametric imaging features with glioma IDH mutations using 3D-CNN trained with 94 patients of IDH mutation, and 120 wild-type gliomas have shown greater success achieving 98% sensitivity, 97% specificity, with an AUC of 99% [51].

#### MGMT promoter

Another important prognostic molecular marker is the methylation of O-6-methylguanine-DNA-methyltransferase (MGMT). The MGMT is an enzyme of DNA repair. Methylation of MGMT deactivates a gene, mediates DNA damage, and dealkylates DNA. This mutation occurs approximately in 33–57% of patients with diffuse glioma. Patients with this mutation have a better prognosis and respond better to alkylating agents (temozolomide chemotherapy) [45, 49]. Several studies have correlated multimodal MRI (T1, T2, FLAIR, and T1C) with glioma MGMT mutation status. Their predictive accuracies ranged from 61 to 80% on cohort ranged from 82 to 193 patients [52–54]. Another study added perfusion MRI to the multimodal MRI and demonstrated important features for identifying MGMT as increased relative CBV in T1C and a higher ratio of contrast-enhancing tumor to complete tumor volume [52]. A radiogenomic study performed with 2D–3D hybrid CNNs achieved 83% accuracy in the prediction of MGMT promoter [50].

#### 1P19Q codeletion

About 30% of low-grade gliomas have 1p19q codeletion of the chromosome arm. 1p19q codeletion is not present in glioblastoma. The presence of 1p19q codeletion with IDH mutant gliomas defines oligodendroglioma. While, the 1p19q non-co deleted tumors are classified as astrocytomas with or without IDH1/2 mutation and have more “circumscribed” margins and display the (T2-FLAIR mismatch) pattern [25, 32, 49]. A study used an SVM classifier, and based on conventional MRI; it allowed classification of IDH mutation and 1p19q codeletion status with 88% and 96% accuracy, respectively [55]. Another study applied CNNs to conventional imaging features and found that increased enhancement, infiltrative margins, and left frontal lobe predilection are associated with 1p19q codeletion with 93% accuracy. Applying CNN helped in automatic lesion and pattern recognition, although an insufficient number of samples to train a reliable model still of technical concern [56].

#### Epidermal growth factor receptor

EGFR is a tyrosine kinase receptor that governs normal epithelial cell growth. The mutations of *EGFR* are found in about 40% of glioblastomas but are seldom present in lower-grade gliomas. The splice variant III (*EGFRvIII*) is the most common *EGFR* mutation in glioblastoma and is found in 31% of patients [49]. Prediction of EGFR has been performed in several radiomic studies using SVM classifiers with approximately 80–85% accuracy in defining EGFRvIII mutation in glioblastoma which exhibits more aggressive features and deep peritumoral infiltration [57, 58]. Another study based on complex multiparametric MRI features has shown increased neovascularity, cell density, and preferential location in the frontal and parietal regions [59].

#### Transcriptomic delineation of glioblastomas (GBMs)

Transcriptomic profiling is a technique used to characterize glioma heterogeneity. It identifies the tumors into four molecular subtypes; classical, mesenchymal, proneural, and neural [27]. A study that applied SVM and based on conventional MRI sequences showed 71% accuracy in delineating the four subtypes [60].

*Other genotypes* Multiple other less commonly associated mutations in gliomas such as *Vascular endothelial growth factor*, *Platelet-derived growth factor* (*PDGF)*, *PTEN* (*Phosphatase and tensin homolog*), *Cyclin-dependent kinase inhibitor (CDKN2A)*, *Proliferating cell nuclear antigen (PCNA)* TERT promoters, and TP53 have also been reported [27].

#### Radiogenomics of oligodendroglioma

The presence of 1p19q codeletion with IDH1/2-mutant gliomas defines oligodendroglioma and is associated with “poorly circumscribed” margins, slight frontal lobe predilection, heterogeneous T1 and T2 signals, and lower ADC values. Oligodendroglioma and astrocytoma have mutations in IDH with TERT promoter and are preferentially located in the medial frontal cortex region. The radiomic features showed high accuracy in discriminating IDH1/2-mutant, IDH1/2-mutant with TERT promoter mutation, and IDH wild type [61].

### Pre-treatment evaluation

#### Tumor segmentation

Malignant tumors have a characteristic growth patterns and tissue changes. Tumor components comprise compartments or “segments” like the solid enhancing portion, necrotic core, non-enhancing tumor, and perifocal edema. Contouring of these segments is essential in the planning of radiotherapy for measuring the gross tumor volume and the clinical target volume, as well as after tumor resection for imaging follow-up [28, 32]. Manual 3-D segmentation methods are time-consuming and subjective. With the advance in computational capabilities, numerous algorithms have been developed and used in semiautomated and automated segmentation methods for brain gliomas [28, 62]. Machine learning has emerged with fast and reliable brain tumor segmentation methods, based on voxel-level classification tasks which differentiate each given voxel whether it belongs to normal brain tissue, glioma, or edema, and extract those features. Currently, segmentation tools are based on DL using CNNs and classifier methods such as SVM and random forests [31, 32, 63]. Recently, CNNs have achieved outstanding performance with about 90% accuracy in voxel labeling [64].

#### Infiltration and extent of brain tumors

Differentiation between tumor infiltration and edema is crucial for pre-surgical planning and safety margin consideration, and it is usually difficult by conventional MRI. Machine learning methods provide a roadmap for evaluating tumor infiltration on preoperative imaging. Features extraction from FLAIR and ADC maps using voxel-wise logistic regression model is able to predict areas of tumor infiltration or future tumor recurrence sufficiently [49, 65]. Some studies used an SVM classifier to register areas of tumor recurrence to preoperative MRI, and based on their features on conventional and advanced MRI, they yielded predictive peritumoral marginal infiltration maps with 90% accuracy [57, 66]. Another study developed an approach to register biopsy sites to preoperative imaging using a CNN method. They used multimodal MRI measures at biopsy sites and applied a network on a cell density counting method to the pathology images. This approach assessed the relationship between cell density and degree of enhancement and generated non-invasive maps of cell density to identify infiltrative tissue margins [67].

### Prognostic value-survival

Currently, poor overall prognostication of tumors is based on independent risk factors like histological grade and clinical models comprising elder age more than 60 years, male gender, functional status of the patient (preoperative Karnofsky scores), partial resection of the advanced tumor, and surgery without chemo-radiotherapy. In addition, molecular markers are important for the diagnosis and prognosis of brain tumors [25, 49]. Previous studies had applied different imaging modalities depending on measuring the maximal dimensions, the volume of enhancing lesion, and white matter tract infiltration. They showed higher predictiveness than clinical models [68–70].

With the advent of AI algorithms, several studies derived radiomics features for predicting the overall survival (OS) of gliomas. A study extracted radiomics features from conventional and advanced MRI metrics including tumor volume, angiogenesis, peritumoral infiltration, and cell density. They used the SVM model to predict the OS of gliomas and classified it into low, medium, and high survival; they achieved 80% accuracy in training and prospective replication cohorts [71]. Another study investigated the effectiveness of two-stage multi-channel 3D deep learning applications on the OS of 68 high-grade glioma patients. The first stage used 3D-CNNs to automatically extract imaging features from multimodal preoperative MRI, DTI, and resting-state functional MRI (rs-fMRI). The second stage added the clinical features including the patient’s age, sex, histological grade, location, and size of the tumor. The deep learning imaging features along with the clinical tumor features were fed into an SVM classifier and achieved 90.66% accuracy in the prediction of long versus short-term OS in high-grade glioma [72].

### Post-treatment evaluation

#### Pseudo-progression (PSP)

Pseudo-progression is defined as an increase in enhancement and/or T2/FLAIR signal abnormality on MRI within 12 weeks after radiotherapy or combined radiotherapy and chemotherapy with spontaneous resolution or stabilization without change in management. Pseudo-progression occurs in 15–50% of patients with gliomas particularly MGMT methylated and IDH mutant tumors undergoing standard therapy. On the other hand, antiangiogenic drugs may induce pseudo-response which means the striking reduction in enhancement due to changing the blood–brain barrier with no or little change in the progression of infiltrating portion [73, 74].

The differentiation of PSP from true progression (TP) is difficult on MRI despite its importance on patient management. Artificial intelligence methods are trying to solve the diagnostic dilemma. A study used conventional MRI, DWI, and PWI for differentiating PSP from TP in 61 glioblastoma patients within 3 months after radio-chemotherapy and surgical resection. The selection of imaging features was done by LASSO logistic regression model. They confirmed that the multiparametric radiomics model had better performance (AUC = 0.90) than any other single parameter. This model should be verified in a multicenter setting independently and prospectively to become a useful tool in the differentiation of PSP from TP [75]. Another study performed on 78 glioma patients, using the hybrid deep and machine learning CNN-LSTM (long short-term memory) method and comprised clinical and imaging features extracted from post-contrast MRI, showed AUC of 0.83 to classify PSP from TP [76].

#### Residual/recurrent tumor versus post-treatment changes

One of the most challenging decisions in the treatment planning of gliomas is the differentiation between tumor recurrence and post-treatment changes. The standard combined treatment of glioma by radiotherapy and chemotherapy usually leads to radiation necrosis which commonly occurs within two years after treatment of glioma. Conventional MRI usually has limited ability in differentiating radiation necrosis from tumor recurrence [77]. Currently, the application of artificial intelligence has been used to classify radiation necrosis and glioma recurrence using either handcrafted radiomics features or deep features alone but they did not fully characterize tumor heterogeneity [78, 79]. A novel study incorporated handcrafted features with deep features on multimodality MRI and constructed logistic regression models. The best model achieved the highest AUC of 0.99, sensitivity of 0.99, and specificity of 0.97 in the validation set with improved performance in the characterization of tumor heterogeneity and classification of radiation necrosis and recurrence [80].

## Radiomics of non-gliomas

### Metastasis

The ability to discriminate glioblastomas from solitary brain metastasis is still challenging, particularly when using conventional MR, due to their similar MR imaging features. Although advanced MRI techniques such as DTI, MR spectroscopy, and perfusion studies have shown important differences between glioblastoma and solitary metastasis, they are not widely used in clinical practice [81–83].

#### Differentiation of glioblastomas from solitary brain metastasis

Currently, a few recent studies used different artificial intelligence techniques to differentiate both entities. A study based on post-contrast 3D T1W gradient-echo sequence radiomics, and data classification using SVM, showed an accuracy of 85% and an AUC of 0.96 [83]. A study based on contrast-enhanced images used multiple feature selection and classification methods like SVM and LASSO. This study constructed radiomics classifiers that showed favorable accuracy and AUC performance of 0.90. Moreover, the clinical performance of the best classifiers was better than expert neuroradiologists [84]. Another study based on DTI metrics (Fractional anisotropy and ADC) and used MR texture analysis showed significantly higher heterogeneity of the peritumoral edema of glioblastoma and metastasis due to its infiltrative nature. This study was limited by its retrospective nature, manual ROI drawing, and absence of histological verification of tumor infiltration [85].

#### Classification of the subtypes of brain metastasis

Classification of the subtypes of brain metastasis was achieved also by radiomics using SVM classifier with high performance, AUC was 0.83, 0.81 for lung and breast metastases, respectively [83]. Another study used quantitative MRI features to discriminate between metastatic subtypes. The selected classifier reached AUCs ranging from 0.64 for non-small lung cancer and 0.82 for breast cancer [86].

### Primary central nervous system lymphoma

#### Differentiation of PCNS from glioma

Primary central nervous system lymphoma (PCNSL) and glioblastoma have very similar conventional MRI visual features that may fail to solve the problem of differentiating both entities. Yet, both are having different management. Therefore, an early and accurate diagnosis is highly important to improve the prognosis. Generally, PCNSL is treated with chemotherapy and whole-brain radiotherapy, whereas patients with glioblastomas commonly undergo gross surgical resection followed by chemo-radiotherapy [87, 88].

Several studies based on extracted MRI features and ADC parameters have reviewed the performance of different ML algorithms as SVM classifier, random forest analysis, or decision tree ML algorithms in the differentiation between PCNSL and gliomas. They showed predicting high-accuracy results up to 96.8% and AUC up to 0.99 [88]. Another study selected the extracted (DTPA) from the first pass of contrast phase of DSC-enhanced perfusion maps and differentiated glioblastomas from PCNSLs [36].

### Hemangioblastomas (HB)

#### Differentiation of HB from brain metastasis

Hemangioblastomas and metastasis are the most common cerebellar masses in adults. Discrimination between the two diseases is important as HBs are benign tumors of vascular origin having good survival rate than brain metastasis. Therefore, their therapeutic approaches and prognosis are quite different [89]. The role of machine learning algorithms in differentiating the two entities is still not established. A recent study performed on a large cohort of intra-axial posterior fossa tumors applied different machine learning algorithms like the random forest, CNN, SVM, and others. The study was based on extracted structural MRI features and ADC histogram analysis. The decision tree model with terminal nodes classified the most common tumor pathologies with 90% accuracy. While random forest models classified the most common 5 tumors including HB and posterior fossa metastasis with an AUC of 0.961 in training datasets, and 0.873 in validation sets [90].

## Radiomics of extra-axial brain tumors

### Meningioma

Meningiomas are the most frequent extra-axial central nervous system tumors. The majority are benign lesions (> 80%) and classified as (WHO grade I), atypical type is < 15% (WHO grade II), and malignant type is < 5% (WHO grade III). Differentiation between histological grades is crucial before treatment decisions. Meningiomas may exhibit intratumoral heterogeneity with variable degrees of vascularity, necrosis, infiltration, and rarely transformation from low to high grade. Conventional MRI remains the standard imaging modality for provisional diagnosis and follow-ups of meningiomas despite lacking the ability to determine the biological behavior and recurrence potential of the tumor [91, 92]. Advanced MRI techniques like DWI and PWI have been applied previously in the diagnosis and grading of meningiomas but with overlapping results [93].

Several studies have introduced machine learning as a radiological tool to improve diagnostic accuracy, prognosis, subtypes, and grading of meningiomas.

#### Grading of meningiomas

Multiple studies have investigated the use of conventional radiomic features as tumor morphology, texture, histogram, and deep learning radiomics to distinguish between low- and high-grade meningioma [94, 95].

Radiomic analysis has shown successful discrimination between meningothelial, fibrous, and transitional meningiomas with achieved accuracy of the validation model as 94.2% [96].

#### Prognosis of meningiomas

An important morphological radiomic feature in the prognosis of meningiomas is the sphericity, previous studies demonstrated that high-grade meningioma tends to be less spherical than low-grade meningioma and is associated with local recurrence and less favorable OS [95, 97].

Regarding the prediction of relapse, a study applied a binary tree model based on extracted radiomic features from T2WI, T1C, and ADC metrics to predict recurrence of skull base meningiomas, the study yielded 90% accuracy [98].

#### Prediction of brain invasion in meningiomas

A study has shown successful preoperative prediction of brain invasion in meningiomas, they applied SVM models derived from T2WI, and T1C images and achieved an AUC of 0.819. The addition of clinical features yielded better predictive performance with an AUC of 0.857 [99].

#### Differentiation of meningioma from craniopharyngioma

A recent study has constructed a binary logistic regression model to distinguish craniopharyngiomas and meningiomas; they achieved an AUC of 0.776 [100].

#### Differentiation of meningioma from hemagioperictyoma

Solitary fibrous tumor/hemangiopericytoma(SFT/HPC) is a rare tumor of vascular origin that became one entity in 2016 WHO classification. Malignant SFT/HPC is WHO grade II and III with aggressive behavior with high rates of recurrence and metastasis [101]. In MRI, SFT/HPC may mimic angiomatous meningioma which is often benign. Consequently, their treatment strategy is largely different, and proper preoperative assessment is crucial [101, 102].

The application of ML has exhibited a promising ability in the differentiation of angiomatous meningiomas from SFT/HPC; a study used SVM models based on textural features derived from T2WI, FLAIR, T1C, and DWI to test their usefulness in differentiating the two tumor entities found that the T1C-based classifier (AUC = 0.90) had significantly better performance than other classifiers [102].

### Pituitary adenoma

Pituitary adenoma is the most common sellar region tumor. Resection through the trans-nasal trans-sphenoidal approach is the preferred technique for macroadenomas as well as functioning tumors. Preoperative assessment by MRI is mandatory to assess tumor morphology, extension, and behavior like invasion of the cavernous sinus. Also, MRI helps in differentiating pituitary adenoma from craniopharyngioma which often shares the same clinical presentation. Proper MRI assessment guides the surgical plan either gross tumor resection or resection followed by neo-adjuvant radiotherapy for adenomas with cavernous sinus invasion. Therefore, an accurate predictive model selecting the most informative radiomic features will be helpful in surgical decision-making [103, 104].

#### Prediction of cavernous sinus invasion

A study aimed to predict cavernous sinus invasion by pituitary adenomas; selected features were based on T1C and T2WI using an SVM classifier with an AUC of 0.826 for the test set [103].

#### Differentiation between pituitary adenoma and craniopharyngioma

A study extracted the qualitative MRI features and texture features from preoperative MRI based on T1C and T2WI to test the difference between pituitary adenoma and craniopharyngioma and demonstrated a significant difference between the tumor entities [104].

#### Prediction of pituitary macroadenomas ki-67 proliferation index

In 2017, the WHO introduced the definition of “high-risk” adenomas that show aggressive behavior and the unpredictable outcome, these tumors are characterized by rapid growth, radiological invasion, and high Ki-67 proliferation marker. A study demonstrated the effectiveness of ML to preoperatively predict the pituitary macroadenomas ki-67 proliferation index class; they extracted features from T2WI from 89 patients, and a k-nearest neighbors (k-NN) classifier was applied to predict macroadenoma high or low proliferation index and achieved 91.67% accuracy in patient’s classification [105].

### Schwannoma

Vestibular schwannoma treatment options differ according to the tumor size. Surgical resection is preferred for large tumors, whereas radiosurgery is usually recommended for small- and medium-sized schwannomas. Gamma knife radiosurgery (GKRS) is effective and safe radiosurgery for controlling tumor growth. Accurate tumor delineation is mandatory before GKRS to identify tumor location, measure tumor volume and detect the tumor response [106, 107].

#### Prediction of prognosis

A study constructed a two-level machine learning model that achieved an accuracy of 85% in the prediction of pseudo-progression after GKRS. This study was based on five radiomic features and showed an inhomogeneous hypointensity pattern of contrast enhancement and variation in T2-weighted intensity [107]. Another study used a series of multiparametric MRI before radiosurgery to capture areas of tumor inhomogeneous intensity like solid enhancing part and cystic part, they proposed an end-to-end DL segmentation scheme and further compared it with the manual segmentation method, with an exceeding accuracy of the AI model to 99% in determining the tumor progression, pseudo-progression, and regression following radiosurgery [108].

## Radiomics of pediatric brain tumors

### Characterization

#### Post fossa tumors

Among pediatric brain tumors, posterior fossa tumors are the most common solid tumors. The most common pediatric posterior fossa subtypes include medulloblastoma, pilocytic astrocytoma, and ependymoma. Differentiation between the subtypes is crucial as each tumor has different management and prognosis [109]. Accurate preoperative diagnosis is required to tailor surgery and drug therapy. Conventional MRI is the key imaging tool for the qualitative assessment of pediatric tumors. Yet, definite tumor histopathological classification is done by biopsy. At present, radiomics and DL methods are developing non-invasive tumor classification models for predicting pediatric posterior fossa tumors [109–111].

A retrospective study that included 288 children with posterior fossa tumors (medulloblastoma, pilocytic astrocytoma, and ependymoma) applied the tree-based automatic pipeline optimization model and extracted the radiomics features from T1C, T2WI, and ADC maps. The automatic radiomics model achieved an AUC of 0.91 and an accuracy of 0.83. Moreover, the tree-based automatic pipeline optimization model achieved significantly higher accuracy when compared with standard manual optimization by a qualitative machine learning expert (0.83 vs. 0.54, *p* = 0.001) for binary classification [112]. Another study investigated a large cohort of 617 children with posterior fossa tumors including pontine diffuse midline glioma, medulloblastoma, pilocytic astrocytoma, and ependymoma, and the study constructed a 2D deep learning architecture model and used T2WI as input for tumor extraction. The performance of the deep learning model was compared with that of four radiologists and yielded an AUC of 0.99 in the accuracy of tumor detection and accuracy of 92% in tumor classification. Despite high accuracy, this study was limited by model overfitting due to the increased number of input data from T1 + C, ADC, and T2. Also, there was a lower scan resolution of ADC images when compared to T1 and T2 images [111].

### Radiogenomics

Table 3 shows the radiogenomic features of the important biomarkers of pediatric brain tumors.

**Table 3:** Radiogenomic features of pediatric brain tumors biomarkers

	Genetic biomarker	Features
1—Medulloblastoma	SHH	
	Wingless-type	~ 90% 5-year survival rate
	Group 3	< 50% overall survival
	Group 4	
2—PLGG	BRAF fusion	Favorable outcome
	BRAF V600E	High risk of progression
3—DMG	H3 K27M mutation	Shorter median survival
	Wild-type H3 K27M	Improved survival

*SHH* sonic hedgehog, *PLGGs* pediatric low-grade gliomas, *DMG* diffuse midline gliomas

#### Medulloblastoma

*Medulloblastoma* is the most common pediatric brain tumor. It was classically thought that medulloblastoma has a single tumor entity. Currently, according to the WHO classification of CNS tumors, four molecular subgroups have been recognized, they are named sonic hedgehog (SHH), wingless-type, group 3, and group 4, each subgroup has its different therapy and prognosis. A favorable outcome is seen with WNT-pathway-activated tumors, and patients have a nearly 90% 5-year survival rate, but patients with group 3 tumors have less than 50% overall survival. Definite tumor subtyping is done with tissue sampling obtained from surgical resection or a single biopsy. However, these are invasive techniques and limited by extensive cost as well [113, 114]. Artificial intelligence is an emerging technology that aims to link imaging features with the tumor molecular subtype [114, 115].

A study included 109 pediatric patients with medulloblastoma applied two predictive test models; a double tenfold cross-validation model and a 3-dataset cross-validation model. Patients underwent molecular analysis from tissue sampling, and the four distinct subgroups were identified. Image data were extracted from T1WI, T1C, and T2WI. The best performance is achieved with the double tenfold cross-validation model for the prediction of SHH, group 3, and group 4 tumors with combined use of T1WI- and T2WI, AUC was 0.79, 0.70, and 0.83, respectively [115].

#### Low-grade glioma

*Pediatric low-grade gliomas* (pLGGs) account for approximately 40% of childhood central nervous system tumors and comprise a heterogeneous form of tumors according to the WHO classification, as grades I or II. PLGGs include juvenile pilocytic astrocytoma (JPA), ganglioglioma, dysembryoplastic neuroepithelial tumor, pleomorphic xanthoastrocytoma, and diffuse low-grade glioma. The standard treatment is surgical excision when possible, but when total resection is not possible, multiple recurrences may occur, and the 10-year progression-free survival is less than 50% [116].

Common alterations occur in the mitogen-activated protein kinase pathway, either fusions or mutations in the B-Raf proto-oncogene, serine/threonine kinase (BRAF) gene, and they have named the BRAF fusion and BRAF V600E point mutation (p.V600E). Recently, it was known that patient prognosis differed in pLGGs according to the molecular alteration. A favorable outcome is seen in patients with BRAF fusion and neurofibromatosis type 1, while those with the BRAF V600E show a high risk of progression and transformation [117, 118].

A recent study investigated a radiomics model to predict BRAF fusion and BRAF V600E mutation. The study included 115 patients with low-grade glioma. Radiomics features were extracted from tumor segmentation and based on FLAIR MRI. The predictive model was tested using a random forest approach for all available tumor types. BRAF status was predicted with an AUC of 0.75 (SD, 0.12) and (95% confidence interval 0.62–0.89) for the internal validation cohort through a fourfold cross-validation scheme, and AUC for the external validation was 0.85. Age and tumor location were significant predictors of BRAF status, while sex was not a significant predictor [119].

#### Diffuse midline gliomas (DMG)

Pediatric diffuse midline gliomas are an aggressive heterogeneous group of brainstem tumors and account for 10% of childhood cancer-related deaths. Previously, these tumors were named diffuse intrinsic pontine gliomas (DIPG). Diffuse tumor location limits its surgical resection. Radiotherapy has been considered the standard palliative treatment. Conventional and advanced MRIs play a major role in the diagnosis and evaluation of tumor therapy response; however, they have no value in predicting patients’ survival [120]. Molecular biomarkers such as H3 K27M mutation status are helpful as tumor histopathology in diagnosing tumors. H3 K27M mutation is an independent predictor of overall survival and is described in midline structures like the thalamus, brainstem, and spinal cord. Patients carrying H3 K27M mutation have a shorter median survival time than patients who have wild-type H3 K27M. Midline gliomas with H3 K27M mutation exhibit different imaging features [121, 122]. Radiomics is a non-invasive method connecting the quantitative features from imaging, such as computed tomography and MRI to the molecular status of the tumors. A study tested the MR textural analysis to predict the OS of pediatric DMG. The study used commercially available TexRAD research software, images extracted from T2 WI and ADC maps. The best predictor stratified the patients into poor and good prognostic groups; the more homogeneous texture is related to the worse prognosis. The median survival was 7.5 months for poor prognosis and 17.5 months for a good prognosis [123].

Another study used the automated machine learning method and included 40 patients with H3 K27M mutations and 60 wild-type patients. Radiomics features were extracted from FLAIR images; the Tree-based Pipeline Optimization Tool (TPOT) was applied to select radiomics features. The model achieved an AUC of 0.903 in the test cohort providing high performance in the prediction of H3 K27M status [121].

## Merits and challenges

### Merits

The deployment of AI in the field of neuro-oncology imaging has many merits. First, it is a fully automated system merged with the radiological workflow for image analysis, quantification, and segmentation that shortens the time consumed in tedious and repetitive tasks, with subsequent improved productivity and efficiency of the clinical workflow for better and quicker diagnosis and decision-making. Second, it could help in image registration with the ability to compare numerous images to track and monitor treatment in real-time. Third, AI can connect patient data such as molecular biomarkers with the non-invasive prediction of neoplasm type or grade and generation of prognostic or predictive models for patient stratification and outcome [3, 8, 49].

### Challenges

Despite of good and promising results, there is still some discrepancy between results in research studies and limited applications in the clinical practice caused by some obstacles. First, the system must be fully integrated well into the radiologist’s workflow. Second, manual segmentation is time-consuming; therefore, application of the semiautomated or fully automated segmentation method is helpful. Third, limited information obtained from previous publications in translating tools and sharing the methodology of this system hinders generalizability [49].

Some important challenges are needed to be considered when building a robust radiologic model including; the generalizability of AI models as most models are built from limited data that is assuming to be representative of all other data in the future for different institutions [124]. One of the premier challenges that developers face is the availability of sufficient data to overcome measurement errors, most annotated data are stored within the hospital systems, sharing of these data among institutions will improve the generalizability [125]. Another crucial challenge is an inter-scanner variability; radio-phenotypic characterization is based on the type of MRI machine, field strength, homogeneity, and acquisition parameters. Harmonizing radiomics can help avoiding the inter-scanner variability by focusing on the radiomics discriminative features and make it reproducible [126].

Most research studies are deficient in validation of their generated models; this hampers the applicability of AI methods [8]. The performance of each model is task-dependent and dataset-dependent. The American College of Radiology Data Science Institute is concerned with standardization and benchmarking of standard use cases that can help in annotation tools and datasets [49]. Another important challenge is the lack of genomic ground truth data which means more tissue biopsy to better prediction and characterization of MRI [25].

## Conclusion

We concluded that AI is a promising tool that combines clinical, radiomics, and molecular markers essential to improve patient’s outcome with differentiation of brain tumors from simulating lesions, characterization, and grading of brain tumors, pre-and post-treatment assessment, and discrimination of tumor recurrence from post-treatment changes. However, many encounter challenges that need much work to be done to deploy AI into daily practice to enhance radiologists’ accuracy and efficiency.

## Data Availability

Data and material are available from the corresponding author upon request.

## Abbreviations

AI: Artificial intelligence
DL: Deep learning
ML: Machine learning
PCNSL: Primary Central Nervous System Lymphoma
